# Impact of universal home visits on maternal and infant outcomes in Bauchi state, Nigeria: protocol of a cluster randomized controlled trial

**DOI:** 10.1186/s12913-018-3319-z

**Published:** 2018-07-03

**Authors:** Anne Cockcroft, Khalid Omer, Yagana Gidado, Adamu Ibrahim Gamawa, Neil Andersson

**Affiliations:** 1 CIET/PRAM, Department of Family Medicine, McGill University, 5858 Cote des Neiges, Montreal, Canada; 2 CIET in Nigeria, Federal Low Cost, Near Police Station, Bauchi, Bauchi State Nigeria; 3 Federation of Muslim Women Association of Nigeria (FOMWAN), Bauchi Chapter, FOMWAN Nursery/Pri/Sec. Schools, AllahiruBatarwa Street, G.R.A., PO Box 2539, Bauchi, Bauchi State Nigeria; 4 Bauchi State Primary Health Care Development Agency, Ministry of Health, Bank Road, Bauchi, Bauchi State Nigeria; 5 Centro de Investigaciones de Enfermedades Tropicales (CIET), Universidad Autonoma de Guerrero, Acapulco, Guerrero Mexico

**Keywords:** Home visits, Maternal and newborn health, Nigeria, Randomized controlled trial, Stepped-wedge design, Edutainment, Male involvement

## Abstract

**Background:**

Maternal mortality in Nigeria is one of the highest in the world. Access to antenatal care is limited and the quality of services is poor in much of the country. Previous research in Bauchi State found associations between maternal morbidity and domestic violence, heavy work in pregnancy, lack of knowledge about danger signs, and lack of spousal communication about pregnancy and childbirth. This cluster randomized controlled stepped-wedge trial will test the impact of universal home visits to pregnant women and their partners, and the added value of video edutainment.

**Methods:**

The trial will take place in six wards of Toro Local Government Area in Bauchi State, Nigeria, randomly allocated into three waves of two wards each. Home visits will begin in wave 1 wards immediately; in wave 2 wards after one year; and in wave 3 wards after a further year. In each wave, one ward, randomly allocated, will receive video edutainment during the home visits. Female home visitors will contact all households in their catchment areas of about 300 households, register all pregnant women, and visit them every two months during pregnancy, after delivery and one year later. They will use android handsets to collect information on pregnancy progress, send this to a central server, and discuss with the women the evidence about household factors associated with higher maternal risks, using video clips in the edutainment wards. Male home visitors will contact the partners of the pregnant women and discuss with them the same evidence. We will compare outcomes between wave 1 and wave 2 wards at about one year, between wave 2 and wave 3 wards at about two years, and finally between wards with and without added edutainment. Primary outcomes will be complications in pregnancy and delivery, and child health at one year. Secondary outcomes include knowledge and attitudes, use of health services, knowledge of danger signs, and household care of pregnant women.

**Discussion:**

Demonstrating an impact of home visits and understanding potential mechanisms could have important implications for reducing maternal morbidity and mortality in other settings with poor access to quality antenatal care services.

**Trial registration:**

Registration number: ISRCTN82954580. Registry: ISRCTN. Date of registration: 11 August 2017. Retrospectively registered.

## Background

A 2015 World Health Organization (WHO) report listed the maternal mortality ratio (MMR) in Nigeria as 814 maternal deaths per 100,000 live births, among the highest rates in the world, with 58,000 maternal deaths every year in the country, and little reduction in maternal death rates between 2005 and 2015 [1]. The situation is even worse in northern Nigeria: the United Nations Population Fund (UNFPA) estimated the MMR in Bauchi State as 1380 in 2006 [2] and a small study reported 621 deaths in 12,067 un-booked deliveries in Bauchi State referral hospital, equivalent to an MMR of 5146 [3].

Ensuring that all women attend for antenatal care (ANC) is proposed as a key strategy for reducing maternal mortality [4]. Routine ANC in Nigeria, as in many other places, is not strongly evidence-based. The poor quality of evidence on ANC is well known, as is the failure of ANC impact studies to control for confounding factors such as socio-economic status, education, maternal age, knowledge, and general access to services [5]. The WHO recommendation for quality aspects of ANC visits [6] is often ignored; four ANC visits to poorly equipped and staffed facilities are not at all the same as four visits to facilities where all recommended services are available.

An analysis based on data from the 2013 Nigerian Demographic and Health Survey reported only 11% of women attending routine ANC received a minimum acceptable quality of care and only 5% the desirable quality of care; in North Eastern Nigeria these figures were 7 and 2% respectively [7]. This stark reality in places like Bauchi makes it unhelpful to say pregnant women should go to the health facilities for ANC. Channelling more women to underfunded services will not improve the already strained quality of care, and may even compromise existing quality of care. Government health services throughout Nigeria, supposedly free for pregnant women, are famously subject to unofficial charges levied on users, as health workers supplement their incomes at the expense of those who need care.

Carroli et al. [5] noted that women who do not attend ANC are usually those most at risk of negative maternal outcomes. A systematic review of 28 studies identified women’s and their husbands’ education, economic status, parity, place of residence, and accessibility to health services as key determinants of use of ANC services in developing countries [8]. A 2009 study in Bauchi reported more ANC use among women with education, from urban areas, who received information from a health worker, who reported help from family members in pregnancy, who had more than two previous pregnancies, whose family owned motorised transport, and whose community had a government ANC facility. Focus groups identified costly, poor quality and inaccessible services, and uncooperative partners as reasons why women did not attend ANC [9].

Maternal mortality is notoriously related to the same “structural factors”: extreme poverty, powerful gender disparities, social marginalisation and low levels of education [10, 11]. Knocking on the door of the home of every pregnant woman to discuss pregnancy risks with her and her spouse can be a structural intervention. It reduces or eliminates the isolation of individual women and, by the content of the dialogue (work in pregnancy, family violence, discussion of pregnancy with spouse and danger signs in pregnancy), provides a strong message about the value of every pregnant woman. Since all households are included, it should be able to reach those who do not attend ANC yet may be at high risk of adverse outcomes.

### Other evidence about the potential impact of the trial intervention

#### Home visits

A body of literature in North America covers home visits to pregnant women by nurses or para-medical staff, often involving higher risk women rather than all pregnant women. Maternal mortality is very low even among higher risk cases in this setting; most studies examine pregnancy outcomes for the child, such as premature delivery and low birth weight. A review of 28 studies concluded that prenatal visits may improve engagement of high risk women and consequently their use of facilities-based prenatal care, but there was limited evidence for an impact on birth weight or length of gestation [12].

A 2010 systematic review of 18 studies of community-based intervention packages in developing countries, including home visits to pregnant women in 15 of the studies, found evidence of a reduction in neonatal mortality and maternal morbidity, but not of a reduction in maternal mortality [13]. A subsequent systematic review examined the impact of birth preparedness and complication readiness interventions on maternal and neonatal mortality in developing countries. Five of 14 trials included home visits, and seven measured maternal mortality. The meta-analysis reported a reduction in maternal mortality when the intervention coverage was more than 30%. The authors recommended further randomized controlled trials (RCTs) of such interventions, particularly in West and Central Africa [14].

#### Mobile technology

This trial applies the gains from work in Giade local government authority (LGA) (see below) in use of mobile technology for data gathering, monitoring and analysis, adapting this as a medium for the video edutainment during home visits. This is an opportunity to address some of the questions from the massive growth in m-health over recent years. Few m-health initiatives ever reach scale implementation [15], and many use systems that are not interoperable (enabling exchange of data among systems in common formats, the use of common protocols, and ultimately the ability to work together) [16].

Systematic reviews of m-surveys and m-surveillance [17–23] report poor quality studies with high risk of bias and only the weakest suggestions of health impact: modest benefit helping patients quit smoking, improving HIV medication adherence, and improving aspects of clinical diagnosis and management. To gather any real momentum in primary health care, the m-health approach needs to show that health impact is possible. The proposed research offers a rigorous evidence base to inform scale-up, with a much needed focus on impact on maternal survival.

#### Video edutainment

There is a growing use of video edutainment in health and use of mass media edutainment to promote behaviour change in public health [24]. Systematic reviews concluded that individual edutainment using video games and other computer technologies improved knowledge, adherence and outcomes in children with diabetes [25, 26]. Edutainment often uses personal stories to communicate messages, although these are not invariably helpful. A review of patient decision aids concluded that addition of personal stories did not help people’s informed decision making [27].

The trial will contribute to the literature about the role of edutainment in health by testing, in a pragmatic cluster randomized controlled trial, the impact on maternal health outcomes (and related intermediate variables) of adding evidence-based video edutainment to home visits during pregnancy.

#### Male involvement in reproductive health

WHO recommends increasing male involvement in reproductive health to improve maternal and child outcomes [4]. A systematic review of strategies to increase male involvement in developing countries, mostly in Asia, found a reduced risk of post-partum depression, improved use of maternal care services, and possibly a reduced risk of childbirth complications [28]. The home visits in the trial in Bauchi will involve the partners of pregnant women as much as the pregnant women themselves.

### Previous work in Nigeria relevant to the trial

This project builds on previous work in Nigeria by the research team with state ministries of health in Bauchi and Cross River states, to support evidence-based planning of health services [29]. Under this program – the Nigerian Evidence-based Health System Initiative (NEHSI)  - in each state, more intensive data collection and dissemination focussed on three randomly selected LGAs. The Bauchi State focus LGAs were Giade, Toro, and Darazo.

The 2009 NEHSI baseline study focused on maternal outcomes and their actionable determinants, and identified four key associations with maternal outcomes: experience of domestic violence in pregnancy, heavy work during pregnancy, lack of basic knowledge of danger signs, and lack of communication with the spouse about pregnancy and delivery [30]. These are areas where increased male involvement in reproductive health could make a difference.

Soap operas are popular entertainment in Nigeria and the NEHSI team shared survey findings with stakeholders through a locally made 20 min video docudrama depicting risk factors and their consequences. A guide helped fieldworkers to facilitate systematic discussion of the issues raised in the video, stimulating discussions with community leaders, community groups, and government health services planners. The docudrama was so well received, especially at community level, that the present proposal tests the value of adding video edutainment to home visits to pregnant women and their spouses.

Under NEHSI, a project run by this research team in Giade LGA in Bauchi State developed realistic and acceptable infostructure for universal coverage of home visits to pregnant women and their spouses, covering all 27,000 households and around 8000 pregnant women per year for three years.

The Giade study examined feasibility, logistics and information flows of universal home visits. In addition to its success in infostructure development, the project generated some preliminary evidence of impact on maternal mortality over three infostructure periods (paper-based, smartphone and android tablet). In the first period there were 12 maternal deaths and 1359 live births, in the second 28 maternal deaths and 4488 live births, and in the third 29 maternal deaths and 5126 live births. These figures correspond to maternal mortality ratios (MMRs: maternal deaths per 100,000 live births) of 883, 624 and 566. Despite this significant time trend and favourable comparison with the rest of Bauchi (although not statistically significant), the MMR at the end of the project was still very high.

There were also other effects of the home visits, contrasting Giade with other parts of Bauchi using a state-wide survey in 2013. Compared with other parts of Bauchi, women in Giade were significantly more likely to know about danger signs in pregnancy, they were twice as likely to reduce heavy work during pregnancy, and they were significantly less likely to suffer intimate partner violence. Complications of pregnancy and delivery were also reduced with universal home visits; women in Giade were less likely to report high blood pressure or bleeding in pregnancy, and less likely to report vaginal tears during delivery or post-delivery infections.

Comparisons with other parts of Bauchi almost certainly underestimate the real impact of the home visits, since the NEHSI project was associated with substantial changes across the whole of Bauchi state over the same period. The results suggest potentially important impacts from the home visits, but important questions remain about implementation of home visits as a means to improve maternal outcomes.

### Research question and objectives

The research questions are:

1. Are universal home visits feasible, acceptable and appropriate in an area of Bauchi State in northern Nigeria?
2. How does adding evidence-based video edutainment affect the feasibility, impact on maternal/infant outcomes, and cost of universal home visits?
3. What is the mechanism of impact of home visits on maternal outcomes?

The objectives of the research, to answer the research questions are:

1. With appropriate government counterparts, plan and implement universal home visits to pregnant women and their spouses in randomly selected wards of Toro LGA;
2. Assess the acceptability and impact of the visits on maternal and infant outcomes, and the added value of video edutainment;
3. Assess the mechanisms of impact on maternal and infant outcomes, and the implementation cost of the home visits and video edutainment;
4. Disseminate the research findings, begin institutionalization in the government health services, and plan with them for scale-up of the home visits program;
5. Strengthen local health systems and build skills in evidence based planning and management of primary health care.

## Methods

### Setting

The study will take place in Toro LGA in Bauchi State, Nigeria. Bauchi state in north-eastern Nigeria has around 5 million residents (4,653,066 in the 2006 census). The main religion is Islam, with prominent Hausa and Fulani ethnicities. Family sizes are large and polygamy is common. The 2013 Nigeria DHS reported 73% of women in Bauchi have no education, compared with 38% nationally [31].

Toro is the largest LGA in the state, with a 2014 population of 437,000 (350,404 in 2006 census). The 2013 NEHSI survey [32] found 47% of women in Bauchi and 61% in Toro LGA reported four or more ANC visits in a government health facility in their last pregnancy; 22% in Bauchi and 27% in Toro had a skilled attendant for their last delivery. Among those who attended ANC in a government facility, 57% in Bauchi and 71% in Toro had to pay in cash or kind. The antenatal care women received at government facilities was limited; only 20% had a minimum functional level of care, including having their blood pressure checked at least twice, urine tested at least once, two doses of tetanus toxoid, iron/folate tablets on each visit, and two doses of anti-malarial prophylaxis [K Omer, personal communication, October 2014]. Some 82% of women in Bauchi and 60% in Toro LGA did not reduce heavy work before the last trimester of pregnancy; 17% in Bauchi and 16% in Toro experienced intimate partner violence (IPV) during their last pregnancy [32].

### Conceptual framework

We use a modified Theory of Planned Behaviour to guide design, methods and analysis [33, 34]. This expands the widely-known knowledge-attitudes-practice (KAP) model [35], criticized for not providing detail about the gap between attitudes and practice [36]. Based on a Theory of Planned Behaviour [37], CASCADA is an acronym for a partial order of intermediates between knowledge and action: Conscious knowledge, Attitudes, Subjective norms, intention to Change, Agency to make the change, Discussion with family, peers and neighbours, and finally Action (the Practice in KAP).

**Fig. 1:**
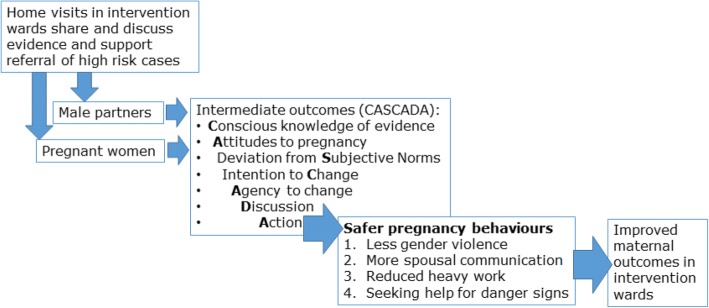
Conceptual framework for behaviour change and outcomes in the target population

The model views all intermediate steps between conscious knowledge and action as likely to be relevant, but not necessarily linearly related; the relative importance of the different steps varies with context. We have used the CASCADA model in resource-poor settings to support analysis and interpretation in cross-sectional studies examining health-related behaviours [38, 39], to guide design of interventions [40–42] and as a framework for qualitative and quantitative analysis of intervention effects [43, 44]. Figure 1 illustrates the way in which, according to the CASCADA model, the home visits will work to improve maternal outcomes.

### Trial design

This is a pragmatic cluster randomized controlled trial (CRCT) in a stepped-wedge design [45, 46], anticipating further rollout after the project and in the meantime allowing us to compare maternal outcomes between wards randomly allocated to receive home visits early with those allocated to receive home visits after a delay (control wards). The two wards in wave 1 will be compared with the two wards in wave 2, and wave 2 wards will be compared with wave 3 wards.

Among wards receiving home visits, one half will be randomly allocated to receive basic home visits and the other half to receive visits plus edutainment (presentation and discussion of evidence-based video clips). We will therefore be able compare maternal outcomes between wards receiving home visits with and without the edutainment element.

### Participants and eligibility criteria

All women of child bearing age in all households (bar none) in the intervention wards will be eligible for the study. Since early marriage is common in the study area, in this study, “child bearing age” is from 14 to 49 years. All women of child bearing age who become pregnant during the study period will be visited at home during their pregnancies, and their husbands will also be visited during the pregnancies. By the end of the study period, all households in all six wards in the study will have received the intervention, with repeated home visits to all pregnant women and their husbands.

### The intervention

Each team of one trained female and one trained male home visitor will have a catchment area of around 300 households, and will visit every household every two months. On the first visit the female visitor will ask about household demographics and socio-economic status. On each subsequent visit, she will check how many women of child bearing age (14–49 years old) are in the household. She will ask each woman of child bearing age about her last pregnancy using the pregnancy history questionnaire. For each currently pregnant woman she will administer a pregnancy surveillance questionnaire. This surveillance questionnaire will be repeated on each two-monthly visit, so a woman contacted early in pregnancy could complete four surveillance questionnaires during her pregnancy. A questionnaire after delivery will record information about the whole pregnancy and delivery, about any maternal deaths, and about birth weight and any neonatal deaths. A final visit one year later will record infant outcomes, including immunisation and episodes of diarrhoea. Male visitors will interview the male partners of the pregnant women, also every two months. The male visitors will not necessarily visit at the same time as the female visitors; many will visit in the evenings or at weekends, when they are more likely to find the male partners at home.

The interviews with pregnant women will include four main topics: domestic violence in pregnancy, heavy work during pregnancy, discussing pregnancy and delivery with spouse, and knowledge about danger signs during pregnancy and delivery. These four items were associated with maternal morbidity in our previous work in Bauchi [30]. For each topic, the visitor will ask about knowledge or experience, share information (for example about danger signs, or about the link between gender violence and pregnancy and delivery complications), and ask what could be done, and is being done, in the home to reduce risk factors (for example to help pregnant women avoid heavy work). The interviews will also ask about attendance for antenatal care check-ups. The interviews with male partners, also repeated two-monthly, will cover the same topics and have a similar format. The instruments will ask about elements of the CASCADA sequence: conscious knowledge of the evidence, attitudes about pregnancy and delivery, deviation from subjective norms, intention to change behaviour, perceived agency to change behaviour, discussion about changes, and action to make changes, as intermediate outcomes in the chain towards safer behaviours and improved maternal outcomes.

The home visits will not routinely encourage pregnant women to attend ANC, but the visitors will be trained to identify high risk cases with reported danger signs, and will refer them to clinics in the area equipped to deal with complications of pregnancy and delivery.

The home visitors will enter responses to interview questions directly into GPS-enabled android handsets, and submit the data to a central server via the mobile network immediately after each interview (or as soon as they reach an area with network coverage). The handsets will also be loaded with information for the female and male home visitors to share with pregnant women and their spouses. And they will be the vehicle for showing the video edutainment in the home visits that include this element (see below). We will use the popular open-source Open Data Kit (ODK) software for the electronic data collection [47].

#### Video edutainment

**Fig. 2:**
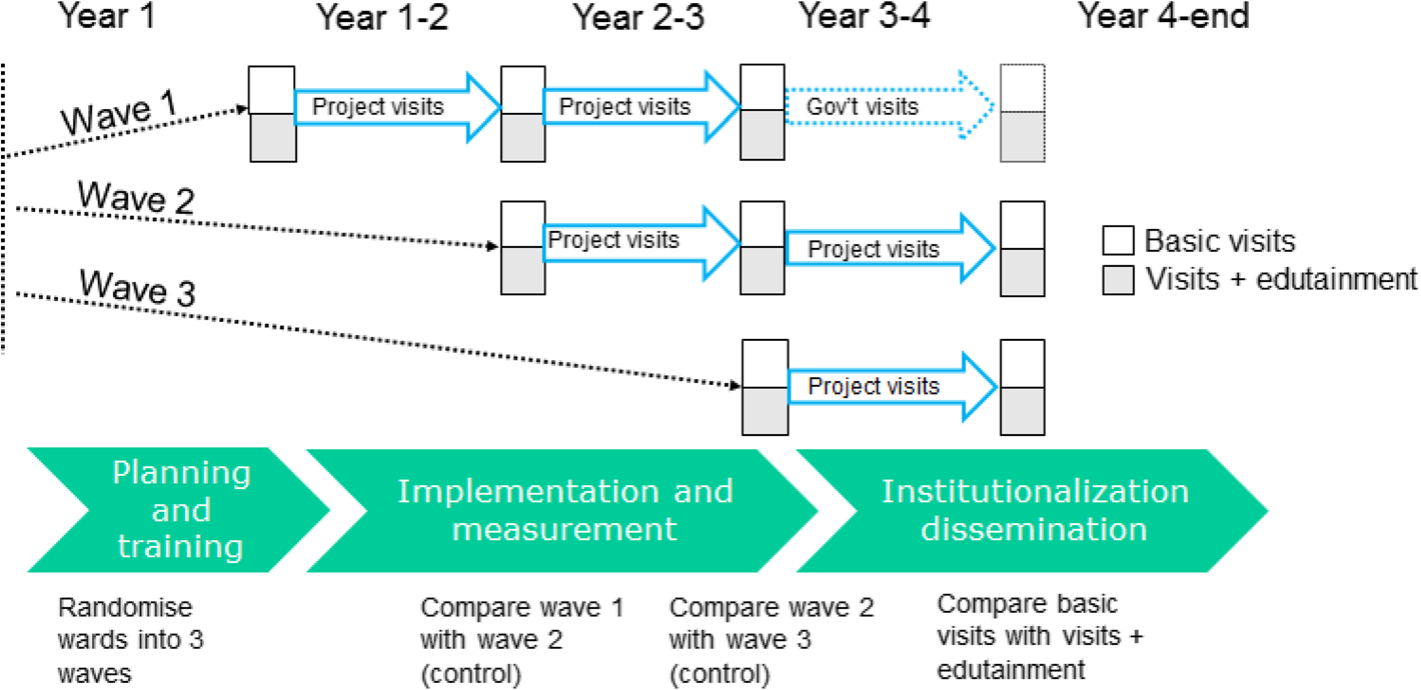
Schematic representation of the study design and timelines. Each square represents a ward

In the planning phase of the study, we will collaborate with local partners to create short videos on the four areas associated with maternal morbidity in our previous work in Bauchi [30]: domestic violence in pregnancy, heavy work in pregnancy, communication with partner about pregnancy and delivery, and knowledge of danger signs during pregnancy and delivery. We will also make short videos about topics related to early childhood health (childhood immunisation, prevention and management of diarrhoea, and breastfeeding), again using evidence from Bauchi [32]. The video clips will be in the form of docudramas, each about two minutes long. In the three wards (one in each wave) randomly allocated to receive the video edutainment, the female and male home visitors will use the video edutainment clips loaded onto the handsets to share evidence with pregnant women and their partners and to stimulate discussion. In the wards not allocated to receive video edutainment, the home visitors will discuss the same topics with the pregnant women and their partners, but without using the video clips (Fig. 2).

### Outcomes

The primary outcomes will be maternal morbidity during pregnancy and delivery reported by women after their latest completed pregnancy (from pregnant women delivering in wards receiving home visits, and from women reporting on their latest completed pregnancy at the baseline visit in the wards not yet receiving the home visits). We will measure rates of severe headaches, swelling of hands and feet, blurred vision, high blood pressure (if measured), problems with urine (if tested), convulsions, and vaginal bleeding during pregnancy. We will also measure rates of reported post-partum high fever and smelly discharge. The study is not powered to detect a difference in maternal mortality between the intervention and control wards but we will nevertheless record and compare maternal deaths between intervention and control wards. Primary outcomes for infants will be pregnancy outcomes (livebirth, stillbirth, miscarriage), birthweight (in terms of the baby being reported as big, normal, or small), immunisation history, and history of episodes of diarrhoea.

Secondary outcomes will be variables in the CASCADA sequence measured in both women and their male partners, including: conscious knowledge about danger signs in pregnancy and about factors related to complications during pregnancy and delivery, attitudes about care for women during pregnancy and delivery, subjective norms about pregnancy and willingness to deviate from unhelpful norms, intention to change behaviours to protect the health of pregnant women, perceived agency to make changes, discussion about pregnancy and delivery between husband and wife, and actual changes in practices reported (particularly in relation to domestic violence and heavy work in pregnancy). We will also analyse any differences in access to health services between pregnant women in intervention and control wards: the proportions who had antenatal care visits to facilities, who had their blood pressure measured, who had their urine tested, who delivered with a skilled attendant, and who delivered in health facilities.

### Timeline

Figure 2 depicts the timelines of the study. At the beginning of the study, we will randomly allocate the six participating wards in Toro LGA into three waves of two wards each. All households in the wave one wards will start to receive home visits immediately. Households in wave two wards will start to receive home visits about one year later, and households in wave three wards will start to receive visits after a further year. By the end of the study period, households in all six wards will be receiving visits. In three of these wards, the visits will include using the video edutainment, while in the other three wards the visits will convey information without the assistance of the video clips.

### Sample size

Our sample size calculations use the clinical trials simulator of Taylor and Bosch [48]. Maternal morbidity and intermediate outcomes in the CASCADA model are common so the study will have power to detect quite small shifts in these outcomes. Our 2013 study found 60% of Toro women did not reduce heavy work in pregnancy and 58% had evidence of post-partum infection or another serious complication of pregnancy [32]. At this level of occurrence, based on an estimated 2880 births in each ward over a two year period, our study will be able to detect a 20% reduction in complications (80% power at the 5% level, k = 0.05) between the home visit wave 1 wards and the next two wards before they start visits (wave 2).

Assuming the visits do reduce post-partum infection by 20%, a comparison of the three wards with visits plus edutainment versus the three with basic home visits would be able to detect a further improvement of 15% (80% power at 5% level, k = 0.05).

The study is not powered to show an impact on maternal mortality, although this should be measurable with later rollout to other LGAs. The Toro MMR of around 800 per 100,000 live births implies around 35 maternal deaths in each ward over a two year period. The wards with universal home visits would have to reduce mortality by 35% to be detected with 80% power at the 5% level (k = 0.06). Assuming the universal home visits reduced mortality risk by this amount, the video edutainment would need to reduce this by a further 60% for this to be detected across three dyads with 80% power at the 5% level.

### Allocation

We will first divide the six wards into two groups, geographically apart. For each wave of two wards, we will randomly select one ward from each of these two groups. The first pair of wards to be selected will be wave 1, the second pair wave 2, and the third pair wave 3. Within each pair of wards, we will randomly allocate one to receive the video edutainment, while the other will receive basic home visits without videos. The home visits intervention will be obvious to households and health services in the wards receiving the visits. We will not attempt any blinding.

### Data collection and analysis

The unit of randomization, intervention and analysis will be the ward. Information on outcomes (maternal morbidity, pregnancy behaviours and care, intermediate CASCADA variables) will come from the questionnaires about last completed pregnancies: from pregnant women delivering in wards receiving home visits, and from women reporting on their latest completed pregnancy at the baseline visit in the wards not yet receiving the home visits. The home visitors will also record maternal deaths.

To measure the impact of home visits compared with no home visits, we will contrast outcomes in wards receiving visits and those not yet receiving visits (wave 1 vs wave 2 in second year; wave 2 vs wave 3 in third year). The first round of home visits in wave 2 and wave 3 will provide the data for these waves as “controls” for the already visited waves (Fig. 2). To assess the impact of the added edutainment in the home visits, we will compare outcomes between the ward with basic visits and the ward with edutainment visits, within each wave. In total, we will compare 3 wards without edutainment with 3 wards with edutainment. We will use generalised estimating equations to incorporate the cluster design and differences at baseline, assuming an exchangeable correlation structure within wards [49].

In addition to the comparisons of proportions of outcomes between visited/not-visited wards and between video/non-video in visited wards, we will also undertake a more detailed analysis of intermediate outcomes in the CASCADA chain. We have previously done this in a cluster randomized controlled trial of community mobilisation for dengue prevention in Mexico [44]. This CASCADA analysis involves transitive closure treating the partial order as a bipolar weighted diagraph [50] with directed edges (arcs) from each factor to each later factor in the order. We convert these to input matrices by rescaling odds ratios from − 1 to 1, using the formula 1-(2/(OR + 1)) and submit these to fuzzy transitive closure using the ProbClosure package [51].

We will estimate the coverage of the home visits program in each ward by comparing the numbers in the submitted data with the population projections from the 2006 census, updated in the baseline household mapping exercise in each ward. We will also measure the proportions of pregnant women who received different numbers of the two-monthly follow up home visits.

We will calculate the total cost of the home visits program, and extrapolate this to estimate the cost of introducing such a program for the whole of Toro LGA, and for the whole of Bauchi state. Using outcomes data, we will attempt to calculate the cost per maternal complication averted, allowing comparison with other programs. We will also calculate the incremental cost of the edutainment implementation strategy.

### Ethics

The trial has approval from the Bauchi State Health Research Ethics Committee (NREC/12/05/2015/12), on 12 May 2015, and from the McGill Faculty of Medicine IRB (A06-B35-15A), on 23 June 2015. We will submit requests for approval of any protocol amendments to these same committees.

#### Consent

The research team in Bauchi will discuss the home visits with the leadership of all communities in the participating wards and get their approval to proceed. Fieldworkers will obtain oral informed consent from all female and male respondents in households for every interview. Using a script, they will explain to respondents the purpose of the visits, the confidentiality of responses, and the respondents’ rights not to answer certain questions or to terminate the interview. They will record the respondents’ informed consent on the android tablet at the beginning of every interview. For respondents under 16 years old, they will also obtain informed oral consent from the parent or guardian.

#### Confidentiality

We will treat all responses from participants as confidential. The home visitors will not record names or identifying information alongside participants’ responses. Data submitted to the server will be treated as confidential; geo-referencing data will be deleted after compilation. The home visitors will conduct all interviews with privacy. The training of home visitors will emphasize that they should not proceed with an interview if they cannot establish privacy or if privacy is breached during the interview. Home visitors will not conduct interviews and discussions in households where the occupants are known to them personally.

#### Identified high risk cases

Some pregnant women will report in interviews that they are experiencing danger signs, such as swelling of hands and feet, headaches, vaginal bleeding, or even convulsions. Training of the home visitors will include this eventuality and the android tablet devices used for recording questionnaire responses will carry algorithms of what to do in different cases. Referral is not straightforward as most local health facilities are not equipped or staffed to handle pregnancy, much less delivery, complications. We will establish mechanisms to deal with pregnant women identified as at risk, including making referral arrangements with clinics in the area equipped to deal with the complications of pregnancy and delivery.

#### Minimizing potential risks to participants

Women who disclose sensitive issues, such as experience of domestic violence, might risk retribution if other household members hear of their disclosure; ensuring privacy minimizes this risk. Discussion of topics such as experience of violence or death of a baby might cause distress. The home visitors are not counsellors but their training will cover handling such situations and they will have contact details for local support services.

#### Potential risks to team members

Female home visitors in Giade LGA did not face any safety problems. Their role and recognition by local leaders made them known and respected figures in the communities. We expect the same in Toro. Nevertheless, training will cover basic precautions to ensure personal safety while working alone in communities.

### Knowledge translation and exchange

The project takes a participatory approach to knowledge translation, involving research users throughout the research process [52]. Families and communities in Bauchi state have already contributed to the research design. Survey findings from Bauchi [30] and community views about these findings were the basis for the discussion guide to be used in the home visits and the development of the evidence-based edutainment video clips.

Initial design workshops with government counterparts will finalise design and implementation. Management meetings during the project with government counterparts will update them on progress, share problems and seek solutions. We will train government officers to train and supervise the home visitors, and to undertake quality control monitoring. In year 3–4, these trained officers will support government running of the home visits program in the two wave 1 wards. Analysis workshops, at State and LGA level, will cover data management, analysis techniques and report generation. Interim results dissemination meetings with local stakeholders will share preliminary findings, and discuss any required modifications to the process of the home visits.

As results become available at the end of the trial, two dissemination workshops will share findings with government and non-government stakeholders in Bauchi State.

The research team will present findings at conferences in Africa and elsewhere, where possible involving research users in this process, and publish articles on the research process and findings in peer reviewed journals.

## Discussion

This project will not on its own change the complex history of Nigeria or remove its well-known problems of service delivery. The project addresses the lack of obstetric skills and facilities only indirectly, by attempting to reduce the need for emergency obstetric care*,* not by improving obstetric care – that is still a desperate need in Toro and many other rural areas in Nigeria. Our emphasis is to reduce the need for services by means of the intervention with households. We expect the trial, with its embedded training of government officers, will consolidate local and state level confidence and skills built in our research projects in Bauchi over the last decade, and help to consolidate the lessons local stakeholders might draw about their own system.

Many of the lessons from this project will be relevant in the rest of Africa, including in countries with better functioning primary health care. We propose to demonstrate that women everywhere, even when they do not have everything in the health system working in their favour, can receive home visits at a time when they are most vulnerable. They can learn from and be strengthened by these visits. And the subsystems needed to establish these visits, to increase their relevance, will be available and tested under less than ideal conditions.

### Potential achievements related to the research

#### Impact of home visits in pregnancy on maternal outcomes

The current literature is encouraging but sparse, particularly in countries with very high maternal mortality, such as Nigeria [17]. Additional evidence of impact and mechanisms of impact on maternal outcomes will contribute to understanding of the potential of universal home visits to improve maternal outcomes. This has implications for reducing maternal mortality in other settings with high maternal mortality.

#### Demonstration of the effect of edutainment

Having gone to the trouble and expense of making home visits, it makes sense they should count as much as possible. If video edutainment increases the impact of home visits on maternal outcomes, it argues for such an approach being widely adopted. Equally, if the video edutainment does not increase impact, then even the marginal extra cost is not justifiable, and this is also useful to know.

#### Unbiased evidence of the value of antenatal screening

Conventional antenatal care is institution-based, so evidence of its value refers only to those who can attend antenatal care in the first place. Attendance cannot easily be randomized. This project takes antenatal education to every doorstep, eliminating the selection bias whereby those at highest pregnancy risk are least likely to attend antenatal care. It will provide valuable evidence of the added value of antenatal screening for danger signs, where previous studies are almost all confounded by the fact that attendance at prenatal classes is not random, but determined by the absence of structural obstacles.

#### Engaging men

Facility-based antenatal care inadvertently converts pregnant women into patients, the responsibility of the health care system, often to the exclusion of the partner. Locating antenatal education and support in the home includes men in every pregnancy. We will measure the behaviour change in men, and intermediate CASCADA variables, related to the home visits.

#### The mobile-technology contribution

Advantages of using the handsets for electronic data collection include: built-in checks to avoid data entry errors and many types of fraud; rapid ongoing review of gathered data (daily downloads of the data from the server) to improve quality control; geolocation to detect and prevent duplicated and fabricated records; frequent reporting of findings to feed into decision support systems for government health service providers; providing a vehicle for presenting video edutainment.

#### The CASCADA model is useful for describing the behaviour change

As a conceptual framework for the research this helped to design the intervention in a way that should be most effective in achieving behaviour change; the research also provides validation and further understanding of the model itself, through secondary analysis of impact on the intermediate CASCADA variables.

#### Health planners in Bauchi adopt home visits as a service delivery strategy

We will document the acceptability and coverage of the home visits run by the government in the wave 1 wards in year 3–4 of the project, and document concrete and costed plans for the home visits program in the state and LGA with a specified timeframe.

### Risks and mitigation strategies

At the beginning of the pilot home visits program in Giade LGA, some home visitors attempted to duplicate and fabricate records of visits. The geolocation system of the android handsets makes this easy to detect, as it allows tracking of location and timing to indicate if visits are taking place to separate households with the expected timing. Based on our experience in Giade we have developed a robust monitoring system, with follow-up and field visits as necessary, and dismissal of fieldworkers as a final sanction. We believe this will control the problem of fraudulent reporting by home visitors.

There may not be adequate services available in local health facilities for pregnant women identified in the home visits as being at high risk of dangerous complications during pregnancy and delivery. In the early stages of the project we will work with government health services in Toro to make referral arrangements for high risk cases, including identifying certain health facilities that are equipped and resourced to deal with such cases.

The biggest single limitation of the project in the Nigerian context is that its impact will be limited to “upstream” determinants. Renovation of facilities and reducing the shortfall in skills and supplies is slow, meaning the simple solution of referring all cases to the facilities will simply not work. There have to be very many *fewer* cases and those referred have to be the ones who really need the extra attention to survive. Even so, there will be obstacles to care, including unofficial payments and inadequate staffing and supplies. The project is not resourced to address this risk, so we are clear the objective is to reduce maternal mortality by influencing upstream determinants.

## Abbreviations

ANC: Antenatal care
CRCT: Cluster randomized controlled trial
GPS: Global positioning system
LGA: Local government authority
MMR: Maternal mortality ratio
NEHSI: Nigerian Evidence-based Health System Initiative
ODK: Open Data Kit
RCT: Randomized controlled trial
UNFPA: United Nations Population Fund
WHO: World Health Organization
